# Periodontitis and Mechanical Forces Modulate Ghrelin Receptor in Periodontal Tissues

**DOI:** 10.1111/apm.70259

**Published:** 2026-09-03

**Authors:** Andressa Vilas Boas Nogueira, Thamiris Cirelli, Maria Eduarda Scordamaia Lopes, Luan Viana Faria, Camila Chierici Marcantonio, Rafael Scaf de Molon, Isis Jordão Pinheiro Ribaldo, Marjan Nokhbehsaim, Sigrun Eick, James Deschner, Joni Augusto Cirelli

**Affiliations:** ^1^ Department of Periodontology and Operative Dentistry University Medical Center of the Johannes Gutenberg University Mainz Germany; ^2^ Department of Diagnosis and Surgery, School of Dentistry at Araraquara São Paulo State University (UNESP) Araraquara Brazil; ^3^ Centro Universitário das Faculdades Associadas de Ensino (UNIFAE), São João da Boa Vista São Paulo Brazil; ^4^ Department of Diagnosis and Surgery, School of Dentistry at Araçatuba São Paulo State University (UNESP) Araçatuba Brazil; ^5^ Section of Experimental Dento‐Maxillo‐Facial Medicine University Hospital Bonn Bonn Germany; ^6^ Department of Periodontology, School of Dental Medicine University of Bern Bern Switzerland; ^7^ Research Center for Immunotherapy (FZI), University Medical Center of the Johannes Gutenberg University Mainz Germany

**Keywords:** bone resorption, growth hormone secretagogue receptor, orthodontic tooth movement, periodontium

## Abstract

Ghrelin signaling, mediated by the growth hormone secretagogue receptor (GHS‐R1a), has emerged as an important regulator of inflammation and bone metabolism. This study investigated GHS‐R1a regulation in periodontal tissues exposed to experimental periodontitis (EP), orthodontic tooth movement (OTM), or both. Human periodontal fibroblasts were stimulated with *Fusobacterium nucleatum* , static tensile strain, or their combination, and GHS‐R1a expression was evaluated by RT‐qPCR. In vivo experiments were conducted in rats subjected to EP, OTM, or combined treatment. Gingival GHS‐R1a expression was assessed by RT‐qPCR and immunohistochemistry, while alveolar bone loss was quantified histometrically. Combined bacterial and mechanical stimulation markedly increased GHS‐R1a expression in periodontal fibroblasts, whereas either stimulus alone produced minimal changes. Similarly, combined EP and OTM induced a pronounced early increase in gingival GHS‐R1a gene and protein expression, which declined over time. Animals exposed to both conditions also exhibited significantly greater alveolar bone loss. These findings demonstrate that periodontal inflammation and orthodontic loading dynamically regulate GHS‐R1a, with early upregulation followed by attenuation during prolonged inflammation. The ghrelin/GHS‐R1a system may therefore contribute to the periodontal response to concurrent inflammatory and biomechanical stimuli and participate in maintaining tissue homeostasis under inflammatory stress.

## 1 Introduction

In recent years, accumulating evidence has suggested that ghrelin, a growth hormone secretagogue receptor ligand (GHRL), is a gastric hormone that stimulates growth hormone secretion via the hypothalamus‐pituitary axis and exerts protective effects against inflammation by modulating multiple signaling pathways [1]. The functional roles of ghrelin and other growth hormone secretagogues (GHS) in the immune system and under inflammatory stress conditions have been studied over the past decades [1, 2]. Both in vitro and in vivo studies have demonstrated that ghrelin acts as a potent anti‐inflammatory mediator and represents an interesting therapeutic candidate for the treatment of inflammatory diseases and injuries (2), as well as playing a role in bone metabolism [3, 4].

Elevated levels of ghrelin and its acylated form have been observed in male patients with chronic periodontitis [5, 6]. Periodontitis is a chronic inflammatory condition with multifactorial origins, driven by dysbiotic bacterial biofilms that progressively damage the supporting structures of the teeth [7]. Previous studies have reported that expression of the active ghrelin receptor (GHS‐R1a) is elevated in the early stages of periodontitis but markedly reduced in more advanced stages compared to healthy tissues, potentially as a result of sustained bacterial challenge, which may exacerbate inflammatory responses and contribute to tissue degradation [8]. *Fusobacterium nucleatum*, a key periodontal pathogen, has been associated with the initial upregulation of GHS‐R1a in periodontal ligament cells [8] and osteoblast‐like cells [9]. Inflammatory stimuli, such as interleukin‐ (IL‐)1β, have also been shown to upregulate GHS‐R1a expression in periodontal cells [10] and osteoblast‐like cells [9]. These previous investigations highlight the anti‐inflammatory properties of GHRL, suggesting that the upregulation of GHS‐R1a in periodontal tissues in response to inflammatory or microbial stimuli may represent a negative feedback mechanism aimed at attenuating the initial inflammatory response in periodontal disease [8, 9, 10].

Although inflammation is a key factor in several diseases, such as periodontitis, it is also an essential component of specific therapies, such as orthodontic tooth movement (OTM). Orthodontic mechanical forces induce aseptic inflammation, which promotes the remodeling of periodontal tissues, balancing osteoclastic activity in compression areas and osteoblastic activity in tension areas [11, 12, 13]. The interaction between orthodontics and periodontics has been extensively investigated [14, 15, 16]. Both fields share inflammation as a common biological factor—while orthodontics induces controlled aseptic inflammation, periodontitis is characterized by a dysregulated inflammatory process associated with periodontal pathogen infections. Orthodontic treatments can enhance periodontal health by correcting occlusal imbalances, facilitating oral hygiene, and reducing occlusal trauma. However, maintaining a healthy, inflammation‐free periodontium is essential during orthodontic therapy, as inadequate plaque control may exacerbate the effects of orthodontic forces on a compromised periodontium [15, 17]. Previous evidence has demonstrated that mechanical loading can modulate the periodontal inflammatory response in periodontitis, leading to increased expression of pro‐inflammatory mediators and receptors [18].

Despite recent advances, a significant gap remains in the literature regarding the regulatory role of hormones and neuropeptides, such as ghrelin, in periodontal inflammatory processes. To date, no study has comparatively assessed the role of ghrelin and its receptor in the periodontium under orthodontic mechanical forces and periodontitis. Given the proposed homeostatic and anti‐inflammatory role of the GHRL/GHS‐R1a system, determining whether receptor expression is maintained, enhanced, or impaired when microbial and mechanical stimuli occur simultaneously may provide important insights into periodontal immune responses, tissue remodeling, and homeostasis during orthodontic treatment [8, 9, 10, 18]. Considering that both processes share overlapping inflammatory mechanisms, elucidating the role of ghrelin and its regulatory mechanisms in these conditions represents a novel and relevant contribution to understanding immune responses and tissue remodeling. In this regard, the present study aims to evaluate GHS‐R1a levels under conditions of OTM, periodontitis, and their combination.

## 2 Materials and Methods

### 2.1 In Vitro Experiment

#### 2.1.1 Cell Culture and Stimulation

Human periodontal cells were isolated from periodontally healthy teeth extracted from six patients either during third molar surgery or for orthodontic purposes (mean age: 21.0 ± 1.6 years; range: 16–31 years; 4 males and 2 females). The study protocol was approved by the Ethics Committee of the University of Bonn (approval number: 043/11), and written informed consent was obtained from all participants or their legal guardians. Fibroblasts were harvested from the middle third of the root surface and cultured in Dulbecco's Modified Eagle Medium (DMEM; Invitrogen, Karlsruhe, Germany) supplemented with 10% fetal bovine serum (FBS; Invitrogen), 100 U/mL penicillin, and 100 μg/mL streptomycin (Invitrogen). Cultures were maintained at 37 C in a humidified incubator with 5% CO_2_. Cells between the third and fifth passages were used for experiments. To mimic periodontal inflammation in vitro, fibroblasts were exposed to *Fusobacterium nucleatum* (strain ATCC 25586) at an optical density of OD_660_ = 0.05. Prior to incubation, bacterial suspensions were prepared in PBS at OD_660_ = 1.0 (equivalent to 1.2 × 10^9^ cells/mL) and subjected to ultrasonic sonication twice (160 W, 15 min each). To simulate orthodontic tooth movement in vitro, periodontal fibroblasts were subjected to static tensile strain of high magnitudes (20%, STSH) for 1 day and 2 days. In addition, cells were also exposed to *F. nucleatum* and STSH simultaneously. Untreated cells served as controls. Three independent experiments were performed, each including triplicate samples for every experimental condition and time point, resulting in a final sample size of *n* = 3 per group at each time point. At the end of the experiments, the RNA of the cells was obtained for the subsequent analysis of gene expression by real‐time PCR (as described above).

### 2.2 In Vivo Experiment

#### 2.2.1 Protocol

The experimental animal model was adapted from our previously validated protocol [18]. While the experimental design has been reported previously, all GHS‐R1a analyses reported in the present study are original and unpublished. All experimental protocols complied with the Animal Research: Reporting of In Vivo Experiments (ARRIVE) guidelines [19] and were approved by the Ethical Committee on Animal Use at the School of Dentistry of São Paulo State University, Araraquara, Brazil (protocol number 23/2012). The sample consisted of 108 adult male Holtzman rats, each weighing approximately 300 g. The animals were kept in labeled plastic cages under controlled conditions, with a 12‐h light/dark cycle and a stable room temperature of 22°C–25°C. They had ad libitum access to water and were fed a standard laboratory diet (Labina/Purina, Ribeirão Preto, Brazil).

The animals were randomly assigned to four experimental groups: control (C, *n* = 27), periodontitis (PD, *n* = 27), orthodontic movement (OM, *n* = 27), and combined orthodontic movement and periodontitis (OMPD, *n* = 27). On Days 3, 7, and 15 following the initiation of orthodontic movement (baseline), nine animals from each group were euthanized at each time point through an overdose of anesthesia. The maxillary jaws were hemisected, and half of the block samples were fixed in 4% paraformaldehyde for 48 h and decalcified in EDTA for histometric analysis. The other half of the blocks had the gingival tissue around the maxillary first molars carefully dissected for extraction of total RNA for RT‐qPCR.

#### 2.2.2 Periodontitis Induction

Five days prior to the baseline, the rats in groups PD and OMPD were anesthetized with intramuscular injections of 10% ketamine hydrochloride (0.08 mL/100 g body weight) and 2% xylazine hydrochloride (0.04 mL/100 g body weight). Cotton ligatures were tied around the cervical region of the upper first molars on both sides, with the knots positioned mesially to induce experimental periodontitis. The ligatures were kept in place for a duration ranging from 8 days to 20 days after placement, depending on the scheduled euthanasia time points.

#### 2.2.3 Orthodontic Tooth Movement

OTM was initiated using a closed‐coil nickel‐titanium spring (Sentalloy, GAC, Dentsply), which delivers a consistent force of approximately 25 g. The spring was attached between the maxillary first molar and the maxillary central incisor. To secure the spring to the central incisors, small grooves were created on their surfaces to stabilize a 0.20 mm stainless steel wire (CrNi, 55.01.208, Morelli, Brazil), which was then covered with a thin layer of composite resin. For attachment to the maxillary first molars, composite resin was applied to the occlusal surface before positioning the spring. In order to eliminate occlusal interference, the lower first molars were extracted, as described elsewhere [18].

#### 2.2.4 Histometric Analysis

Nine hemimaxillae from each group were analyzed after 15 experimental days to assess morphological changes. Histometric analysis was conducted by a single, blinded examiner who had been previously trained and calibrated for the specific objectives of the study. Serial parasagittal sections, each 4 μm thick, were obtained and stained with hematoxylin and eosin (HE). Two representative sections per tooth were selected for analysis. Histological images were captured using a Leica DMLS microscope (Leica DMLS; Leica/Reichert Diastar Products & Jung, Wetzlar, Germany) at 200 × magnification. The histometric evaluation was carried out using image analysis software (ImageJ version 1.37b; National Institutes of Health, Bethesda, MD, USA), focusing on the furcation bone area, which was measured within a 1000‐μm region beneath the furcation in the inter‐radicular area of the maxillary first molars.

#### 2.2.5 Immunohistochemistry

Immunohistochemical analysis to evaluate Ghrelin receptor protein within the periodontal tissues was performed by the avidin‐biotin‐peroxidase (ABC) method using the LSAB kit (Dako, Glostrup, Denmark), according to the manufacturer's instructions. Tissue sections with 4 μm thickness mounted on silanized slides (Perfecta, São Paulo, Brazil) were used. Antigen retrieval was achieved by incubating the sections in Rodent Decloaker reagent (Biocare Medical, Concord, CA, USA) using a Decloaking Chamber (Biocare Medical) at 80°C for 30 min. Endogenous peroxidase activity was blocked using a 5% hydrogen peroxide solution, and nonspecific binding was minimized by incubating the sections in 5% bovine serum albumin (BSA; Sigma) diluted in phosphate‐buffered saline (PBS). The slides were then incubated with a rabbit polyclonal anti‐ghrelin receptor antibody (1:100 dilution; ab85104, Abcam, Cambridge, UK). Immunolabeling was visualized using DAB chromogen (Dako), followed by counterstaining with Carazzi's hematoxylin. For each section, six images were captured under an optical microscope (LEICA Microsystems GmbH, Wetzlar, Germany) at 400 × magnification. Positive cells were quantified within a rectangular area of interest (AOI) measuring 66,074 μm^2^ by a blinded and previously calibrated examiner.

#### 2.2.6 Quantitative Real‐Time PCR (RT‐qPCR)

Total RNA was isolated from gingival tissues obtained from nine hemimaxillae per group, at each time point, using the RNeasy Mini Kit (Qiagen), following the manufacturer's protocol. The concentration and purity of the extracted RNA were assessed with a spectrophotometry (NanoDrop ND‐2000, Thermo Fisher Scientific, Waltham, MA, USA). Subsequently, 500 ng of total RNA was reverse transcribed into complementary DNA (cDNA) using the iScript Select cDNA Synthesis Kit (Bio‐Rad Laboratories, Munich, Germany), according to the supplier's instructions. Quantitative analysis of GHS‐R1a and GAPDH gene expression was performed by real‐time reverse transcription polymerase chain reaction using the CFX96 Touch Real‐Time PCR Detection System (Bio‐Rad Laboratories). Reactions were carried out with SYBR Green PCR Master Mix (SsoAdvanced Universal SYBR Green Supermix, Bio‐Rad Laboratories) and gene‐specific primers (QuantiTect Primer Assay, Qiagen). The thermal cycling conditions included an initial denaturation step at 95°C for 5 min, followed by 40 cycles consisting of denaturation at 95 C for 10 s and annealing/extension at 60°C for 30 s. Gene expression levels were quantified using the comparative Ct (ΔΔCt) method.

### 2.3 Statistical Analysis

The GraphPad Prism 9.0 software (La Jolla, CA, USA) was used for statistical analysis and construction of graphical interfaces. The Shapiro–Wilk and Bartlett's tests were performed to check the normality and homoscedasticity of the data. In the presence of the assumptions, comparisons were made using analysis of variance (ANOVA) and Tukey's multiple comparisons test. Kruskal‐Wallis test complemented by Dunn's multiple comparison tests were performed as nonparametric alternatives. Confidence intervals were built around the mean values, considering a 95% confidence level. The significance level adopted was 5% for all analysis.

## 3 Results

### 3.1 Regulation of GHS‐R1a by *F. nucleatum* and Biomechanical Forces in Periodontal Fibroblasts

**FIGURE 1:**
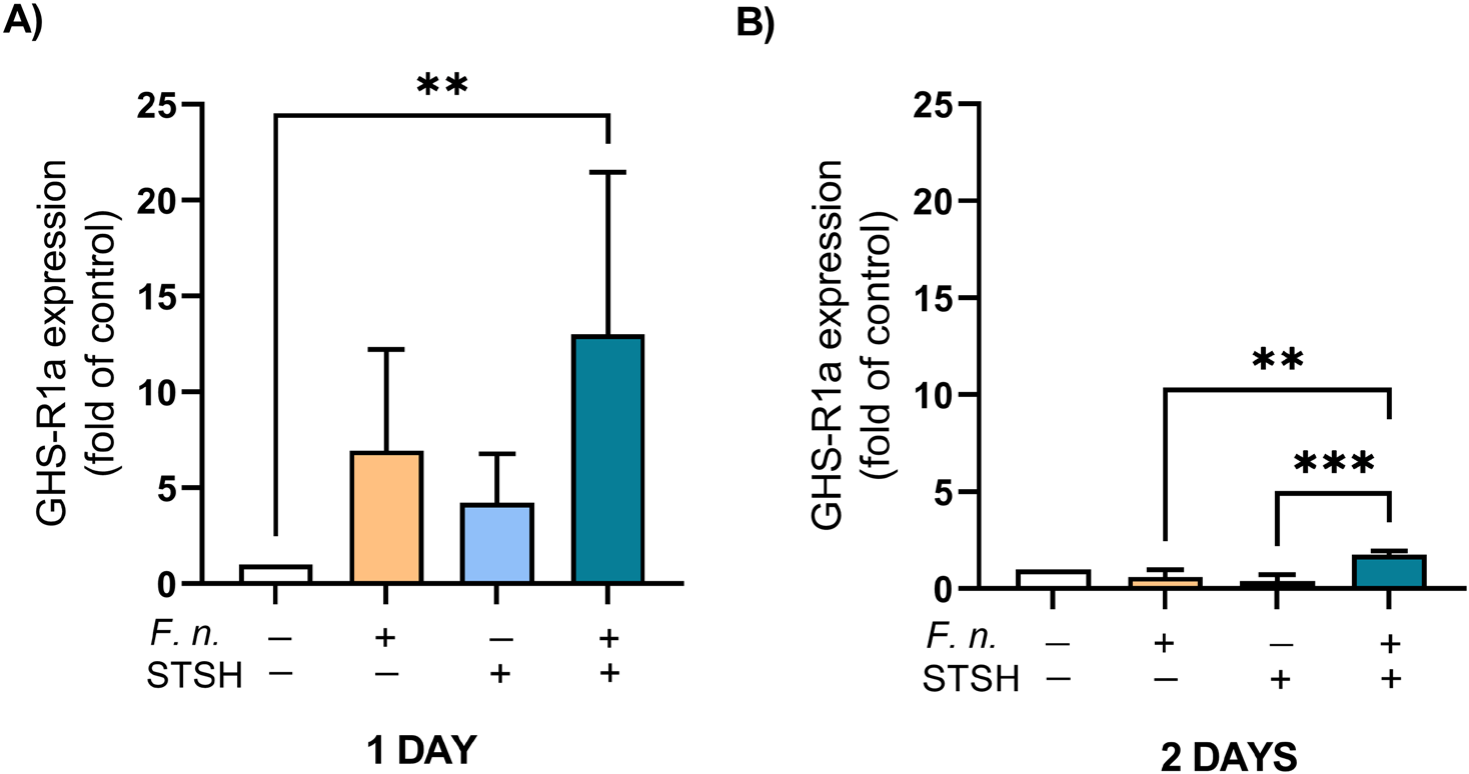
Mean and standard deviation of GHS‐R1a gene expression in fold change in the presence of *F. nucleatum* and/or biomechanical loading in periodontal cells at 1 day (A) and 2 days (B) (*n* = 3 independent experiments per group/time point; samples were analyzed in technical triplicate, with triplicate measurements averaged for analysis). **p ≤ 0.01; ***p ≤ 0.001 by Kruskal‐Wallis test and Dunn's multiple comparisons test.

To investigate the interaction between microbial and biomechanical signals in a controlled environment, an in vitro model using periodontal fibroblasts was employed. As shown in Figure 1A, exposure to *F. nucleatum* alone did not induce a significant increase in GHS‐R1a expression at either of the analyzed time points. A similar effect was observed following biomechanical loading alone, which resulted in expression levels statistically comparable to the control group. In contrast, the combined application of microbial stimulus and biomechanical forces led to a marked upregulation of GHS‐R1a, with an increase of 13‐fold (SD 8.4) after 1 day (*p* < 0.05; Figure 1A), indicating a synergistic effect in the inflammatory events when both stimuli were applied simultaneously.

A similar pattern was observed after 2 days (Figure 1B), with *F. nucleatum* or biomechanical loading alone producing no significant changes in GHS‐R1a expression, whereas their combined application resulted in an even greater induction of receptor expression, which was significantly higher than both the mechanical force and the bacterial stimulation alone (*p* < 0.001 and *p* < 0.01, respectively).

### 3.2 Regulation of GHS‐R1a in Rat Periodontal Tissues With Experimental Periodontitis and/or Orthodontic Tooth Movement

**FIGURE 2:**
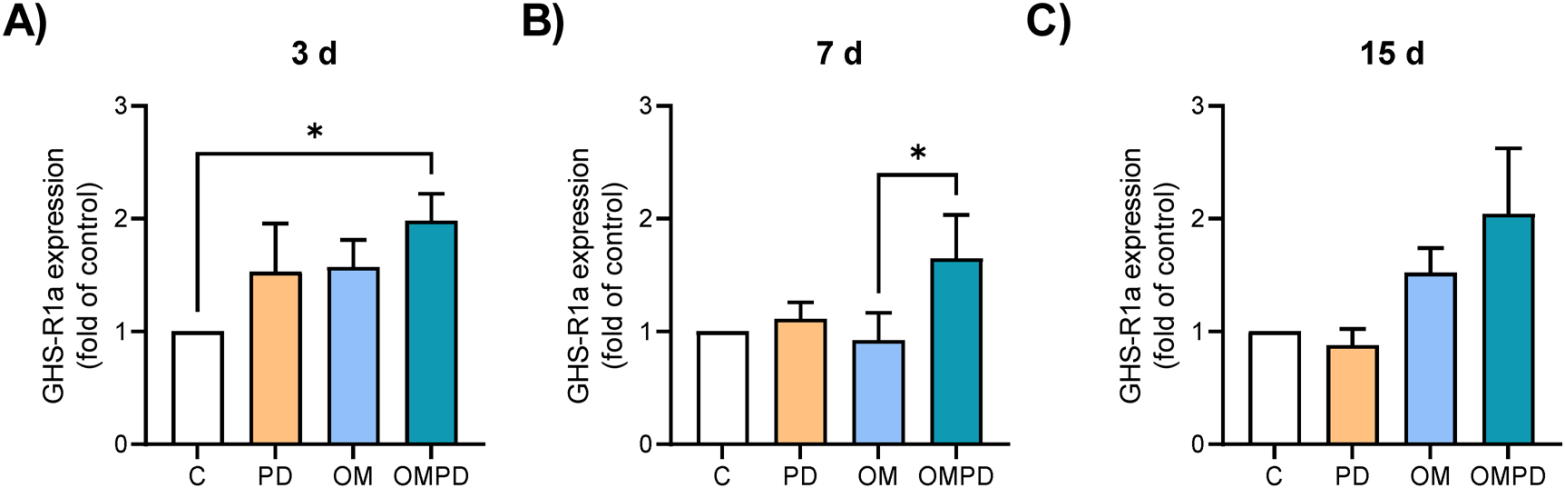
Mean and standard deviation of GHS‐R1a gene expression in fold change compared to the control in periodontal tissues at 3 days (A), 7 days (B), and 15 days (C) (*n* = 4/group/period). *p ≤ 0.05; **p ≤ 0.01 by Kruskal‐Wallis test and Dunn's multiple comparisons test.

Based on the in vitro findings and the increased receptor expression in response to the combined stimuli in periodontal cells, an in vivo investigation of microbial and biomechanical signals was conducted in a model that more closely resembles clinical conditions. EP and OTM models were employed in rats, with untreated animals serving as controls. As shown in Figure 2A, both periodontitis and orthodontic movement alone led to a slight increase in GHS‐R1a expression. However, these changes were not statistically significant compared to the control group (*p* > 0.05). Notably, when sites with EP were also subjected to OTM, GHS‐R1a expression was significantly increased in periodontal tissues at 3 days and 7 days (*p* < 0.05; Figure 2A,B). Interestingly, in the group subjected to both stimuli (OMPD), receptor expression declined over time, reaching levels comparable to the control by Day 15 (Figure 2C).

### 3.3 Elevated Levels of Ghrelin Receptor Protein in Periodontal Tissues of Rats With Experimental Periodontitis and/or Orthodontic Tooth Movement

**FIGURE 3:**
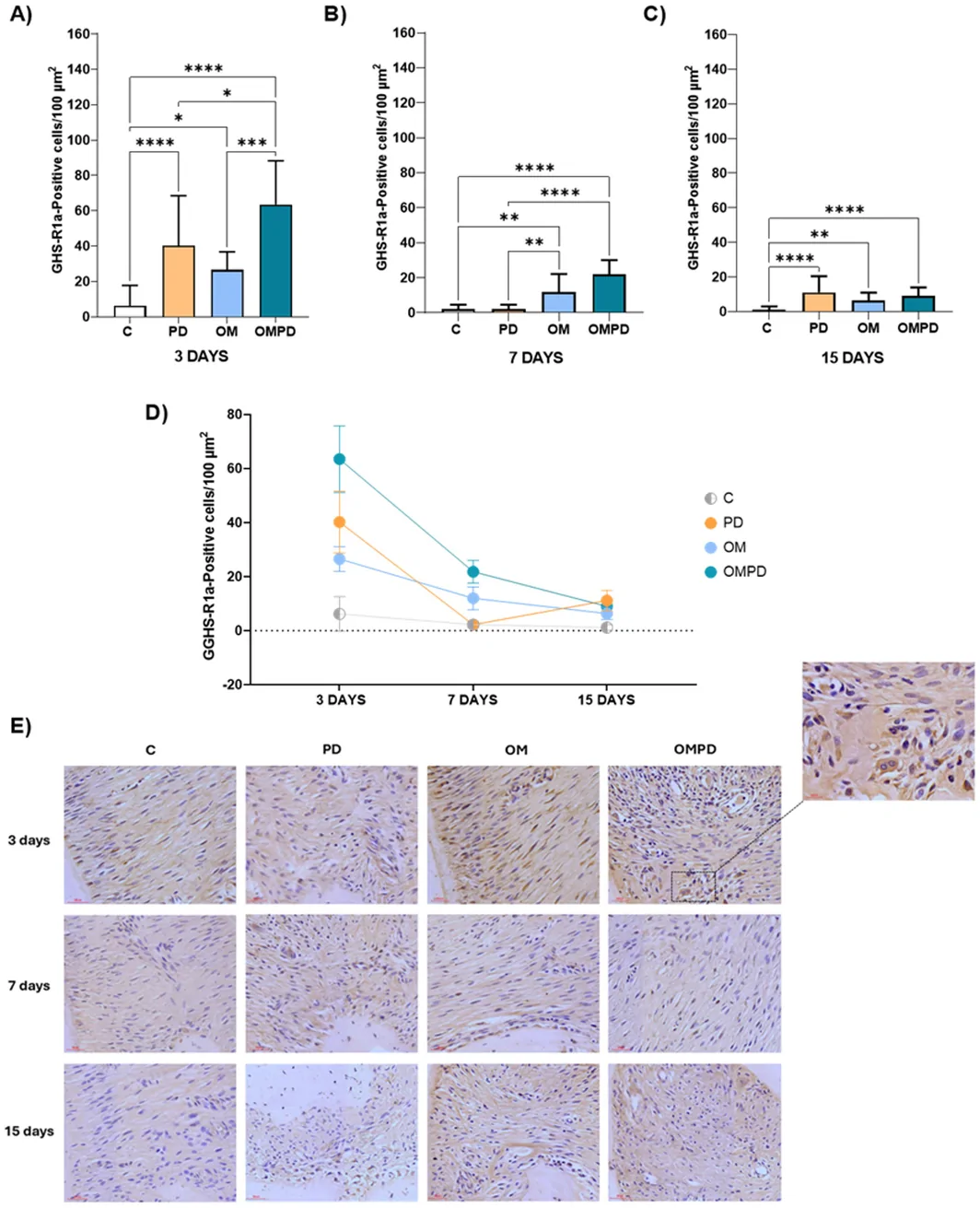
Mean and standard deviation of immunohistochemical staining of GHS‐R1a (positive cells/100 μm^2^) in periodontal tissues at 3 days (A), 7 days (B), and 15 days (C). *p ≤ 0.05; **p ≤ 0.01; ***p ≤ 0.001; ****p ≤ 0.0001 by Kruskal‐Wallis test and Dunn's multiple comparisons test (*n* = 8/group/period). In (D), the mean with 95% confidence intervals is presented for the different time points. (E) Representative images of each experimental group (400 × magnification) according to the analyzed period. On the right side of the panel, a representative 800 × magnification image showing labeled cells.

The protein expression of the ghrelin receptor was evaluated by immunohistochemistry in rat maxillary tissues. Compared with the control group, GHS‐R1 protein expression was significantly (*p* < 0.05) upregulated in periodontal tissues exposed to EP, OTM, and their combination at 3 days and 15 days (Figure 3A,C). However, on Day 7, GHS‐R1a levels in the EP group were similar to those in the control, whereas both the OTM and the combined groups maintained significant (*p* < 0.05) elevated expression levels (Figure 3B). Notably, on Day 3, the combination of periodontitis and tooth movement resulted in a more pronounced increase in GHS‐R1a expression compared to either condition alone (Figure 3A, *p* < 0.05). On Day 7 there were no significant differences between OM and OMPD groups (Figure 3B). By Day 15, GHS‐R1a levels had equalized across all experimental groups (Figure 3C). Representative immunohistochemical images (Figure 3E) corroborate the quantitative analysis, demonstrating weak GHS‐R1a immunostaining in the control group and increased numbers of positively stained cells in the EP, OM, and especially the OMPD groups during the early (3 days) experimental period. The higher‐magnification image further illustrates the cellular localization of GHS‐R1a‐positive cells within the periodontal tissues.

### 3.4 Changes in Bone Structure

**FIGURE 4:**
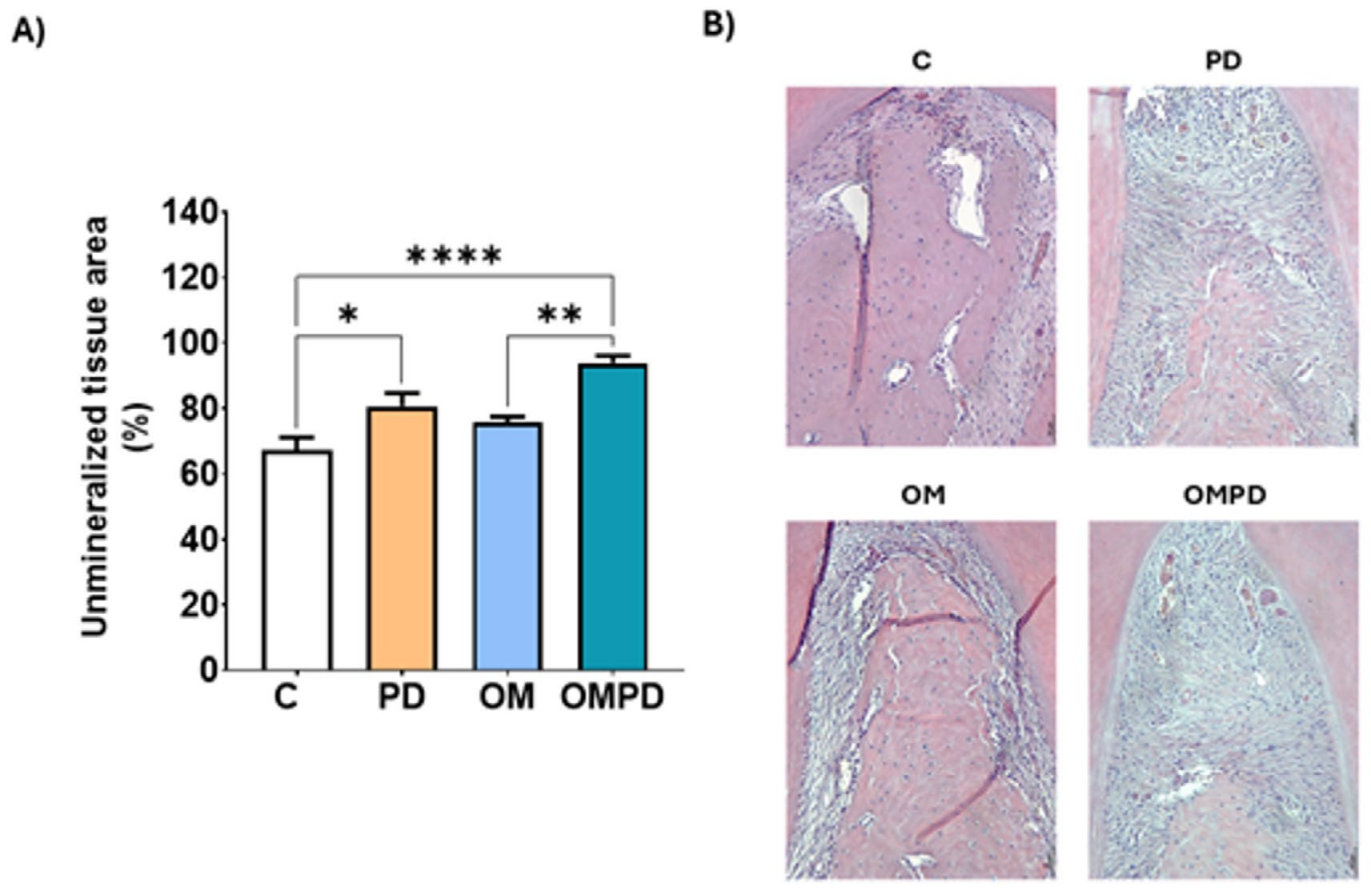
(A) Mean and standard deviation of measurements of alveolar bone loss (non‐mineralized tissue area) in the inter‐radicular area of the maxillary first molars (*n* = 8/group/period). *p ≤ 0.05; **p ≤ 0.01; ***p ≤ 0.001; ****p ≤ 0.0001 by ANOVA and Tukey's multiple comparisons test. (B) Representative images of each experimental group (200 × magnification).

Animals in the EP groups (PD and OMPD) exhibited significantly greater bone loss compared with the control group (*p* = 0.027 and < 0.0001 for PD and OMPD, respectively). No significant difference in bone loss was observed between the C and OM groups. Notably, microbial challenge combined with movement further increased bone loss relative to the OM group, resulting in the largest area of nonmineralized tissue among all groups without periodontitis (C and OM) (*p* < 0.05) (Figure 4A).

Representative histological sections (Figure 4B) corroborate the quantitative findings, showing preserved inter‐radicular bone architecture in the C and OM groups, whereas the PD group exhibited evident alveolar bone resorption and enlargement of the non‐mineralized area. The OMPD group displayed the most pronounced periodontal destruction, characterized by extensive loss of inter‐radicular bone and the largest non‐mineralized tissue area.

## 4 Discussion

The present study provides novel evidence that GHS‐R1a expression is modulated by the combined action of periodontal inflammation and mechanical loading in periodontal cells and tissues. The simultaneous exposure to microbial and biomechanical stimuli induced a stronger early increase in GHS‐R1a than either stimulus alone, both in vitro and in vivo, indicating a synergistic response during the initial phase of inflammation. This effect was accompanied by greater alveolar bone loss in the combined experimental condition, suggesting that GHS‐R1a is involved in the periodontal tissue response to concurrent inflammatory and biomechanical challenges.

It is well established in the literature that orthodontic forces can exacerbate periodontal destruction by intensifying the local inflammatory response [18, 20]. In the present study, we investigated the impact of the combination of two inflammatory stimuli—OTM and microbial challenge—on the modulation of ghrelin receptor expression in periodontal tissues. These findings are consistent with prior studies indicating that the expression of the functional ghrelin receptor in periodontal cells and tissues is modulated by periodontitis‐associated microorganisms [8, 9, 21]. Furthermore, our results provide novel evidence that mechanical stimuli, either alone or in combination with microbial challenges, regulate the protein levels of this receptor. During the early phase, the combination of microbial challenge and orthodontic force led to an upregulation of GHS‐R1a expression. However, prolonged exposure of periodontal tissues to both periodontopathogens and orthodontic force resulted in GHS‐R1a transcript levels comparable to those observed in the control group in the in vivo model. Although GHS‐R1a protein levels remained statistically different, they approached control levels after 15 days in vivo. This overall temporal pattern of GHS‐R1a protein expression suggests that the enhanced response observed during the early phase progressively attenuates as the inflammatory stimulus persists.

The sustained loss of ghrelin's anti‐inflammatory activity in chronic infections may contribute to the exacerbation of the inflammatory response and the progression of periodontal tissue destruction [8]. This may represent one of the underlying mechanisms that explain the greater alveolar bone loss observed in our findings. These results are consistent with previous investigations [8, 10] and support the hypothesis that prolonged exposure of periodontal cells and tissues to inflammatory stimuli leads to a progressive impairment of the protective feedback mechanism, thereby compromising the homeostatic and anti‐inflammatory role of the GHRL/GHS‐R1a system in periodontal tissues.

Recent evidence indicates that mechanical forces modulate the periodontal inflammatory response to disease by upregulating multiple pro‐inflammatory mediators and receptors, consequently promoting increased bone resorption [18]. In the present model, OTM alone did not significantly increase alveolar bone loss compared with the control group. However, when orthodontic forces were applied in the presence of experimental periodontitis, bone loss was significantly greater, reinforcing the concept that the periodontal inflammatory environment may modify the tissue response to mechanical loading. The clinical relevance of our findings becomes particularly evident in the context of orthodontic treatments, which often extends over several months or even years and involves the continuous application of mechanical forces, resulting in sustained bone remodeling [13, 22]. Under such prolonged conditions, exacerbated periodontal inflammation may represent a therapeutic risk, leading to irreversible periodontal tissue loss and additional complications, such as root resorption [23, 24] and endodontic involvement [25, 26]. These risks may be further amplified if anti‐inflammatory mechanisms—such as the GHRL/GHS‐R1a system—are impaired or undergo progressive desensitization over time.

Previous investigations conducted by our group have indicated that orthodontic forces can modulate the expression of pro‐inflammatory and anti‐apoptotic molecules induced by bacterial stimuli [20, 27, 28, 29]. Depending on the molecular profile assessed, the interaction between periodontal inflammation and mechanical loading can enhance the synthesis of certain mediators, while others may be downregulated. These findings reinforce the concept that orthodontic forces extend beyond their classical role in promoting bone remodeling, acting as active modulators of the host immunoinflammatory response to the periodontal microbiota.

Taken together, the present results demonstrate that the interplay between periodontal inflammation and orthodontic forces influences the expression of the functional ghrelin receptor, suggesting a relevant role for this receptor in the regulation of the immunoinflammatory response during prolonged bone remodeling. The reduction in GHS‐R1a expression observed under chronic inflammatory conditions combined with sustained mechanical loading may impair the protective mechanisms of the GHRL/GHS‐R1a system, contributing to the progression of periodontal tissue destruction. Considering the extended duration of orthodontic treatment, understanding these mechanisms is essential to minimize therapeutic risks and improve clinical predictability, particularly for patients with a history of or increased risk for periodontal disease.

Beyond periodontal tissues, the role of the GHRL/GHS‐R1a system has been extensively investigated in other chronic inflammatory conditions, reinforcing its relevance as a regulator of the immune response. In rheumatoid arthritis, ghrelin levels are reduced, and receptor activity attenuates synovial inflammation via PI3K/AKT/NF‐κB inhibition [30]. Similarly, in inflammatory bowel disease, ghrelin administration alleviated colitis in aged mice, decreasing inflammatory markers such as IL‐17A and promoting tissue repair through PPARγ‐dependent metabolic regulation [31]. In sepsis‐induced acute lung injury, ghrelin exerts potent protective effects by suppressing NF‐κB activation, reducing pro‐inflammatory cytokines, and improving survival in experimental models, an effect later confirmed and mechanistically expanded by evidence showing additional inhibition of iNOS and Akt pathways in alveolar macrophages [32, 33].

This broader evidence highlights the translational significance of our findings. Although periodontal disease is not the primary clinical focus for targeting the ghrelin receptor, the consistent involvement of the GHRL/GHS‐R1a system in chronic inflammatory and tissue‐destructive conditions suggests a broader regulatory role in host responses [34, 35]. More generally, the GHRL/GHS‐R1a system is increasingly recognized as a pleiotropic modulator of inflammation, energy balance, and tissue repair across multiple organs [34]. These findings underscore the potential of GHS‐R1a as an adjunctive therapeutic target and suggest that enhancing ghrelin signaling may help mitigate long‐term risks in patients exposed to sustained mechanical and inflammatory challenges [34, 36], including those undergoing orthodontic treatment.

In summary, our findings highlight that the interplay between periodontal inflammation and orthodontic forces modulates the expression of GHS‐R1a, suggesting a relevant role for this receptor in regulating the immunoinflammatory response during prolonged bone remodeling. The reduction in GHS‐R1a expression under chronic inflammatory conditions, combined with sustained mechanical loading, may compromise the protective actions of the GHRL/GHS‐R1a system, thereby contributing to the progression of periodontal tissue destruction. While these results provide valuable insights, they are based on a specific experimental model and selected molecular marker and thus should be interpreted within this context. A deeper understanding of these mechanisms—supported by broader molecular analyses and complementary experimental approaches—may help minimize therapeutic risks and improve clinical outcomes, particularly in orthodontic patients with a history or increased susceptibility to periodontal disease.

The results of this study demonstrate that the modulation of GHS‐R1a is influenced by the interaction between periodontal inflammation and orthodontic forces. This receptor appears to play a significant role in regulating the immunoinflammatory response during force‐induced bone remodeling. These findings contribute to a deeper understanding of the molecular mechanisms involved in periodontitis associated with orthodontic tooth movement.

## Funding

This work was supported by Fundação de Amparo à Pesquisa do Estado de São Paulo, 2010/07771‐4, 2011/13752‐5, 2014/20715‐7, 2017/07137‐2. German Research Foundation, DFG, Germany, DE 1593/5‐1, DE 1593/1‐2. Coordenação de Aperfeiçoamento de Pessoal de Nível Superior, CAPES, ID 88881.144012/2017‐01. German Academic Exchange Service, DAAD, Germany, ID 57391253.

## Ethics Statement

This study was approved by the local Ethical Committee on Animal Experimentation (protocol number 23/2012) and performed in accordance with the guidelines from the National Council for the Control of Animal Experimentation (CONCEA) and by the Ethics Committee of the University of Bonn (approval number: 043/11).

## Conflicts of Interest

The authors declare no conflicts of interest.

## Data Availability

The data that support the findings of this study are available from the corresponding author upon reasonable request.
